# Physiologically based modelling of tranexamic acid pharmacokinetics following intravenous, intramuscular, sub-cutaneous and oral administration in healthy volunteers

**DOI:** 10.1016/j.ejps.2021.105893

**Published:** 2021-09-01

**Authors:** Zoe Kane, Roberto Picetti, Alison Wilby, Joseph F. Standing, Stanislas Grassin-Delyle, Ian Roberts, Haleema Shakur-Still

**Affiliations:** a Quotient Sciences, Mere Way, Ruddington, Nottingham, United Kingdom; b Great Ormond Street Institute of Child Health, University College London, London, United Kingdom; c Clinical Trials Unit, London School of Hygiene & Tropical Medicine, London, United Kingdom; d 3 Hôpital Foch, Suresnes, and Université Paris-Saclay, UVSQ, INSERM, Infection et inflammation, Montigny le Bretonneux, France

**Keywords:** Pharmacokinetics, Physiologically based pharmacokinetic modelling, Tranexamic acid, Intravenous, Oral, Intramuscular

## Abstract

**Background:**

: Tranexamic acid (TXA) is an antifibrinolytic drug that reduces surgical blood loss and death due to bleeding after trauma and post-partum haemorrhage. Treatment success is dependant on early intervention and rapid systemic exposure to TXA. The requirement for intravenous (IV) administration can in some situations limit accessibility to TXA therapy. Here we employ physiologically based pharmacokinetic modelling (PBPK) to evaluate if adequate TXA exposure maybe achieved when given via different routes of administration.

**Methods:**

: A commercially available PBPK software (GastroPlus®) was used to model published TXA pharmacokinetics. IV, oral and intramuscular (IM) models were developed using healthy volunteer PK data from twelve different single dose regimens (*n* = 48 participants). The model was verified using separate IV and oral validation datasets (*n* = 26 participants). Oral, IM and sub-cutaneous (SQ) dose finding simulations were performed.

**Results:**

: Across the different TXA regimens evaluated TXA plasma concentrations varied from 0.1 to 94.0 µg/mL. Estimates of the total plasma clearance of TXA ranged from 0.091 to 0.104 L/h/kg, oral bioavailability from 36 to 67% and Tmax from 2.6 to 3.2 and 0.4 to 1.0 h following oral and intramuscular administration respectively. Variability in the observed TXA PK could be captured through predictable demographic effects on clearance, combined with intestinal permeability and stomach transit time following oral administration and muscle blood flow and muscle/plasma partition coefficients following intra-muscular dosing.

**Conclusions:**

: This study indicates that intramuscular administration is the non-intravenous route of administration with the most potential for achieving targeted TXA exposures. Plasma levels following an IM dose of 1000 mg TXA are predicted to exceed 15 mg/mL in < 15 min and be maintained above this level for approximately 3 h, achieving systemic exposure (AUC0–6) of 99 to 105 µg*hr/mL after a single dose. Well-designed clinical trials to verify these predictions and confirm the utility of intramuscular TXA are recommended.

## Highlights

• Tranexamic acid (TXA) as a treatment for post-partum haemorrhage (PPH) depends on early intervention and rapid systemic exposure.
• This study uses PBPK modelling to evaluate the pharmacokinetics of TXA given by different routes of administration; intravenous, intramuscular, sub-cutaneous and oral.
• Intramuscular administration of 1000 mg TXA is predicted to achieve >15 mg/L in plasma in <15 min and exceed this level for approximately 3 h post dose.

## Graphical abstract

**Figure:**
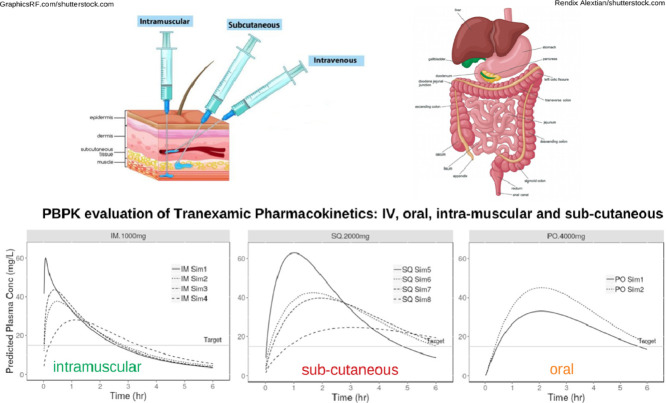

## 1 Introduction

Acute severe bleeding is a leading cause of death (Wang et al., 2015). Traumatic extracranial haemorrhage, often the consequence of road traffic crashes or violence, is responsible for more than two million deaths each year (Bruns and Hauser, 2003). Traumatic intracranial bleeding are common causes of death and disability with an estimated 69 million new cases each year (Dewan et al., 2018). Postpartum haemorrhage (PPH) is a leading cause of maternal mortality and morbidity. About 6% to 10% of all women giving birth develop PPH and it accounts for around 100,000 maternal deaths every year (Calvert et al., 2012; Carroli et al., 2008; World Health Organization et al., 2015). Ninety-nine percent of deaths from PPH are in low and middle-income countries (LMICs) (Say et al., 2014). Many women who survive experience severe morbidity. Some women need surgery to control the bleeding and many require a hysterectomy, thus removing the possibility of having more children.

Tranexamic acid (TXA) is a synthetic analogue of the amino acid lysine, which inhibits fibrinolysis by blocking the lysine binding sites on plasminogen. TXA reduces bleeding by inhibiting the enzymatic breakdown of fibrin blood clots (McCormack, 2012). Plasminogen produced by the liver is converted into the fibrinolytic enzyme plasmin by the tissue plasminogen activator (tPA). Plasminogen and tPA bind to lysine residues on fibrin leading to localised plasmin formation and fibrin cleavage (McCormack, 2012). TXA is a molecular analogue of lysine that inhibits fibrinolysis by competing with fibrin for the lysine binding sites in plasminogen. TXA inhibits the capacity of plasminogen and plasmin to bind to fibrin, hence preserving blood clots from plasmin-mediated lysis (McCormack, 2012).

TXA has been shown to reduce the risk of death from trauma and PPH. The WOMAN trial assessed the effects of intravenous TXA in 20,060 women with PPH (Shakur et al., 2017). TXA significantly reduced death due to bleeding with no adverse effects. When given within three hours of birth, TXA reduced death due to bleeding by nearly one-third (relative risk (RR)=0.69, 95% CI 0.52 to 0.91; *p* = 0.008). However, for many women, treatment is too late to prevent death from PPH. Most PPH deaths occur in the first hours after giving birth (Shakur et al., 2017). In both trauma and PPH, every fifteen minutes treatment delay reduces the survival benefit by about 10% until around three hours after which there is no benefit (Gayet-Ageron et al., 2018). One of the main barriers to rapid treatment is the need for an intravenous (IV) injection. IV TXA can rapidly achieve therapeutic blood concentrations of >10 mg/L, the concentration above which substantial inhibition of fibrinolysis has been observed (Picetti et al., 2019).

If TXA could be given intramuscularly for trauma, it could be given by trained first responders, police officers, ambulance drivers, and primary care nurses with important reductions in time to treatment. Intramuscular TXA might also increase access to treatment for women with postpartum haemorrhage (PPH). In low- and middle-income countries, about 40% of women deliver at home. Although community health workers are often present, most cannot give IV drugs. Transport to hospital can take hours, and many women exsanguinate on the way (Kironji et al., 2018) .Although IV TXA is the treatment of choice, this is not an option for tens of thousands of women. Finding alternative to IV TXA administration in women with PPH is a WHO research priority (Vogel et al., 2018). To facilitate this, developing understanding on how alternative routes to IV TXA administration could impact the pharmacokinetics (PK) is needed.

The objective of this study was to use published literature data to develop a physiologically-based PK model that predicts the plasma concentration-time (Cp-time) profile of TXA following IV, oral (PO) and intra-muscular (IM) dosing. After the TXA model was defined and validated, simulations were performed to predict if appropriate systemic concentrations of TXA could be achieved rapidly enough by either oral, IM or sub-cutaneous (SQ) administration. This work builds on the meta-analysis carried out by Grassin-Delyle et al. (2019)

## 2 Methods

### 2.1 Search strategy

Advantage was taken of the search conducted for a previously published systematic review on the pharmacodynamics of TXA (Picetti et al., 2019). This search contained terms related to PK and all routes of administration. That search was complemented with an additional search until June 2018 for this work. Inclusion criteria comprised any studies conducted in healthy volunteers which reported the PK of TXA administered via the following routes: intravenous, oral (ingestion or sublingual), intraosseous, intra-muscular, sub-cutaneous or transdermal. Two reviewers independently assessed records to determine whether they met the inclusion criteria. Titles and abstracts were screened, and the full texts of any potentially relevant reports were assessed for inclusion. Disagreements between reviewers were resolved by consensus.

### 2.2 Data extraction and handling

PK data sources and key study information are summarised in Table 1. TXA pharmacokinetic data was either manually transcribed or digitally extracted from the selected source publications using DigitTM version 1.0.4 (a plot digitizer tool from Simulations Plus Inc., Lancaster, CA, USA). Where concentration-time (Cp-time) data is reported for 3 (or less) subjects the individual subject data has been directly transcribed into GastroPlus. Where Cp-time data in more than 3 subjects is reported an arithmetic mean Cp-time profile has been calculated and this average profile used during modelling. To aid data visualization all model predictions and simulation results have been exported from the modelling software and summary plots have been constructed using R version 3.4.4.

**Table 1:** PK data sources and key study information.

Source	Route	Fed/Fasted	Dose (mg)	Duration of sampling (h)	Sex/N	Ethnicity	BW (kg)	Age (Yrs)
Eriksson (1974)	IV	na	1000	8	M/2	European	67,69	37, 29
Pilbrant (1981)	IV	na	1000	32	M/3	European	66,80,73	39,43,36
	PO	Fed/Fasted	2000	6	M/10	European	Not reported	Not reported
Puigdellivol (1985)	IV	na	500	8	M/3	European	Not reported	23,25,33
	IM	na	500	8	M/3	European	Not reported	23,25,33
Sano (1976)	IV	na	1000	6	M/2	Japanese	77,75	48,51
	PO	Fed	500	6	M/5	Japanese	58,67,67,54,52	24,44,37,44,48
	IM	na	500	6	M/3	Japanese	77,75,65	48,51,39
Sindet-Pedersen (1987)	PO	Fasted	1000	8	M/5, F/5	European	54 - 90 (□ = 70)	23 - 29 (□ = 25)
Chang (2004)	PO	Fasted	500	12	12	Chinese	Not reported	Not reported
Validation (XP12B-101)	IV	na	1000	36	F/26	American	Not available	Not available
	PO	Fasted	1300	36	F/26	American	Not available	Not available

Model development datasets: Chang et al., 2004; Eriksson et al., 1974; Pilbrant et al., 1981; Puigdellivol et al., 1985; SANO et al., 1976; Sindet-Pedersen, 1987.

Model validation dataset: NDA 022,430 study XP12B-101, Xanodyne Pharmaceuticals Inc., 2009.

### 2.3 Pharmacokinetic model development

The modelling reported here has been performed using GastroPlus® (version 9.5, Simulations Plus Inc., Lancaster, CA, USA) a commercially available PBPK software. Where demographic data was not reported in the source reference, the default GastroPlus® Population Estimates for Age-Related (PEAR) settings have been used. Where body weight was not recorded default weights of 86.3, 69.1 and 61.7 kg, were assumed for European/American, Chinese and Japanese subjects respectively. When demographic information provided included age but not body weight, the default PEAR body weight for the observed age was used. TXA is known to be primarily excreted unchanged in the urine with overall renal clearance equalling total plasma clearance (Pilbrant et al., 1981). Glomerular filtration rate (GFR) as calculated by GastroPlus® is dependant on the ethnicity, gender, age and body weight defined in the PEAR physiology file. Within this TXA model, all plasma clearance is achieved through renal filtration (CLfilt) which has been defined as equal to the calculated glomerular filtration rate (GFR). The GastroPlus® PEAR physiology settings used are shown in Table 2, a European PEAR physiology was not available, so the American settings were applied for European subjects. Where measured in vitro data was not available to inform parameter estimates for essential physical chemical properties, ADMET® version 7.2 (Simulations Plus Inc., Lancaster, CA, USA) was used to predict these properties from structure.

**Table 2:** PEAR physiology file settings .

Source	Route	Population	Weight (kg)	Age (Yrs)	Gender	GFR (mL/s) 2
Eriksson, Pilbrant	IV	American	71	36	M	1.919
Pilbrant	PO	American	86.27	30	M	2.005
Puigdellivol	IV, IM	American	85.12	27	M	2.038
Sano	IV	Japanese	76	50	M	1.763
Sano	IM	Japanese	72	46	M	1.797
Sano	PO	Japanese	60	39	M	1.876
Sindet-Pedersen^1^	PO	American	70	25	M^1^	2.052
Chang	PO	Chinese	69.14	30	M	1.707
Validation (XP12B-101)	IV, PO	American	75.22	30	F	2.005

1 Sindet-Pederson dosed 5 M and 5F subjects but due to software constraints in the PEAR file all subjects specified as male.

2 GFR (mL/s) calculated by GastroPlus based on built in regression models and specified weight, age and population.

**Fig. 1:**
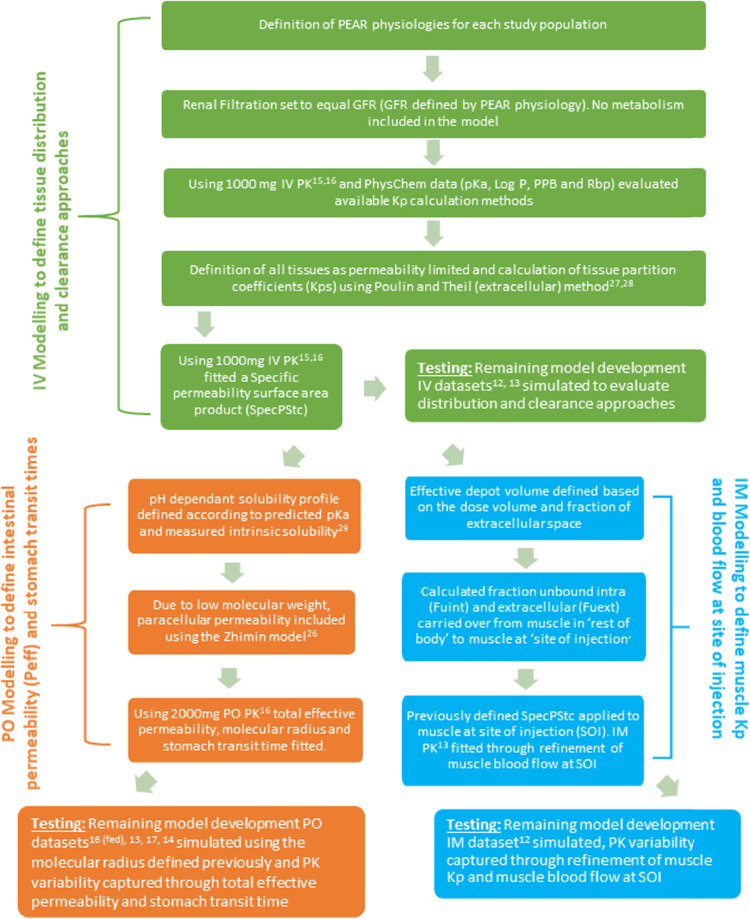
Tranexamic acid model development strategy. Figure key: 12-(Puigdellivol et al., 1985), 13-(Sano 1976), 14-(Chang 2004), 15-(Eriksson et al., 1974), 16-(Pilbrant et al., 1981), 17-(Sindet-Pedersen 1987), 26-(Zhimin 1995), 27-(Poulin 2000), 28-(Poulin 2009), 29-(Pharmacia and Upjohn 2020). PBPK model development strategy.

See Fig. 1 for a flow chart describing the model development strategy.

All tissues within the whole body PBPK model have been specified as permeability limited and tissue partitioning coefficients (Kp's) were calculated using the Poulin and Theil extracellular method (Poulin and Theil, 2009, 2000). Because of the low molecular weight of TXA, paracellular permeability was included in the model and calculated using the Zhimin method (Zhimin, 1995). To allow the observed PK variability following oral and IM dosing to be described certain critical absorption parameters in the intestine and at the site of injection were fitted to the observed PK profiles and allowed to vary across the model development datasets.

### 2.4 Pharmacokinetic model validation

Intravenous and immediate release (IR) oral TXA PK profiles taken from a Lysteda registration study; NDA 022430 study XP12B-101, were used as an external model validation dataset (Xanodyne Pharmaceuticals Inc., 2009).

During model development the key sources of IV and PO PK variability were identified. To allow the external validation data set to be predicted ‘typical’ estimates for these key parameters were defined as follows:

All subjects in study XP12B-101 (Xanodyne Pharmaceuticals Inc., 2009) were female however no body weight or age information for the individual participants was available. The American female PEAR physiology (30 years old, 75.2 Kg) along with the core model parameters (Table 3) defined the clearance and distribution of TXA enabling prediction of the IV validation PK (1000 mg).

**Table 3:** Core model parameters.

Parameter	Abbreviation	Units	Value	Source
pKa / Solubility Factor	pKa / SF	na	Acid 4.51 / 7.8	Predicted from structure
			Base 10.28 / 7.8	
Lipophilicity	Log P	na	−1.79	Predicted from structure
Molecular Weight	Mw	g/mol	157.21	Predicted from structure
Ref solubility at pH 7	Ref Sol	mg/mL	50	Measured 1
Blood to plasma ratio	Rbp	na	1	Fitted
Plasma protein binding	PPB	%	3	Measured 1
Molecular Radius	MR	Å	4	Fitted
Specific Permeability surface area product	SpecPStc	mL/s/mL	1.081×10^–5^	Fitted

1 (Pharmacia and Upjon Co, 2020).

An average effective human permeability (Peff) and fasted stomach transit time (STT) were calculated using the respective model development parameters estimates, these average estimates were then used along with the American female PEAR physiology (30 years old, 75.2 Kg) and core model parameters (Table 3) to predict the oral validation PK (1300 mg fasted state).

### 2.5 Pharmacokinetic simulations

Single dose oral, IM and SQ simulations were performed and evaluated against a target plasma concentration of 15 mg/L, the time to exceed this concentration was also considered. A simulation time of 6 h was employed for all administration routes.

The default American female PEAR physiology was used in all simulations; 30 years old, 75.2 kg.

Oral simulations were performed assuming administration of a solution formulation of TXA. Since solution formulations are known to gastric empty more quickly than solid dosage forms a STT of 0.1 hour was employed in all oral simulations.

For the other critical absorption parameters found to describe the observed PO and IM PK variability estimates informed by model development were used when performing simulations.

## 3 Results

Six publications were identified as eligible for inclusion in this study, providing the data used for model development (Chang et al., 2004; Eriksson et al., 1974; Pilbrant et al., 1981; Puigdellivol et al., 1985; SANO et al., 1976; Sindet-Pedersen, 1987), while data from the Lysteda regulatory filing; study XP12B-101, NDA 022430, was used for model validation (Xanodyne Pharmaceuticals Inc., 2009). All publications were in English, except for one in Japanese (SANO et al., 1976) that was translated with the help of a Japanese native speaker. In total, 12 different TXA PK regimens were evaluated (10 used in model development and 2 in model validation) in which 74 participants (of which 42% were female) received tranexamic acid (TXA), individual study demographics are presented in Table 1. Across the model development data sets plasma concentrations ranged from 0.2 to 94.0 µg/mL, 0.2–14.5 µg/mL and 1.3–21.1 µg/mL following IV, oral and IM dosing respectively. For the method validation datasets plasma concentrations ranged from 0.1 to 90.4 µg/mL after 1000 mg IV and from 0.1 to 12.9 µg/mL after a 1300 mg oral dose.

TXA (4-(Aminomethyl)cyclohexanecarboxylic acid) is a low molecular weight (157.21 g/mol) zwitterionic compound and aqueous solubility and plasma protein binding are reported to be 167 mg/mL in water (Merck Index Online, 2020), >50 mg/mL at pH 6.5–8.0 and 3% bound respectively (Pharmacia and Upjohn Co, 2020). Visych et al. (2016) reports a Caco-2 average permeation rate of 12.4 × 10–06 ± 0.1 cm/s, (Visych et al., 2016) however no marker data is published alongside thereby making the result of limited use in PBPK model development. The effective human permeability (Peff) predicted from structure using the ADMET predictor software is 0.75×10–04 cm/s. No other relevant measured physical-chemical information was found in the literature.

Table 3 summarises all core (fixed across all datasets) model parameters, highlighting if parameters were measured, predicted from structure or fitted using the model development PK datasets.

Using NCA of the model predicted IV Cp-time profiles evaluated in this study, the average total plasma clearance (CLp) and average volume of distribution at steady state (Vss) of TXA are estimated to be 0.10 L/h/kg (range: 0.091 to 0.104 L/h/kg) and 0.59 L/kg (range: 0.546 – 0.613 L/kg)

**Fig. 2:**
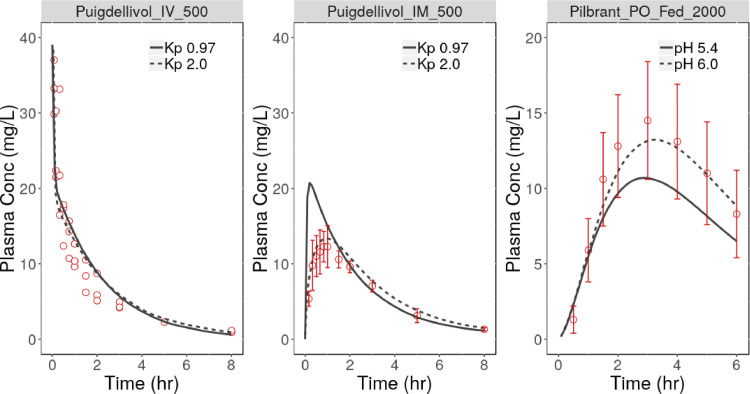
Visualisation of i) the impact of muscle tissue partition coefficient (Kp) on the predicted concentration time profile following IM and IV dosing (left and middle panel) and ii) the impact of fed state jejunum (J1) pH following oral dosing (right panel). The open red circles represent the observed data from the Puigdellivol (ref 12) and Pilbrant (ref 16) study, the solid black and dashed lines represent predicted plasma concentration time profiles. (For interpretation of the references to colour in this figure legend, the reader is referred to the web version of this article.)

Variability in the oral PK across studies was primarily captured through variability in permeability and stomach transit time, see Table 4. Estimates of Peff ranged from 0.38 to 0.50×10–4 cm/s (average = 0.45×10–4 cm/s) across the model development studies evaluated, and STT ranged from 0.38 to 0.75 h in the fasted state (average = 0.54 h). A stomach transit time of 1.0 hour adequately described the two fed state datasets evaluated. However, when modelling the Pilbrant fed state data, (Pilbrant et al., 1981) an increase in the default Jejunum pH was required (J1 compartment pH increased from 5.4 to 6.0) because of the significant impact that intestinal pH had on the amount of predicted paracellular permeability, and therefore overall fraction absorbed (Fa), see Fig. 2 (right panel). Using the default fed state J1 pH of 5.4 a small negative food effect (FE) is predicted (approx. 20% reduction in AUC compared to fasted state) and this was not observed by Pilbrant (Pilbrant et al., 1981).

**Table 4:** Permeability and stomach transit time parameter estimates associated with oral PK modelling.

Parameter	Pilbrant	Sindet-Pedersen	Sano	Chang	Average	Source
Permeability x 10–4 (cm/s)	0.4	0.38	0.5	0.5	0.45	Fitted
Fasted STT (h)	0.5^1^	0.75^1^	na	0.38^2^	0.54	Fitted^1^ / Default^2^
Fed STT (h)	1	na	1	na	1	Default

na: not applicable.

Only 2 IM PK datasets were identified, and the IM specific model parameters used to describe the observed variability in IM PK are summarised in Table 5.

**Table 5:** Muscle blood flow and partition co-efficient parameter estimates associated with IM PK modelling.

Parameter	Sano	Puigdellivol	Source
Muscle Q at SOI (mL/min/100 g muscle)	0.82	1.37	Fitted
Muscle Kp	0.97^1^	2^2^	Calculated^1^ / Fitted^2^

Kp values are calculated based on pKa, Log P, PPB%, Rbp and specific permeability surface area product.

combined with the Poulin and Theil extracellular tissue partition co-efficient (Kp) estimation method.

Both IM datasets were simulated using the minimum calculated depot volume of 42.4 mL. Blood flow (Q) at the site of injection was found to be a very influential parameter on the predicted IM PK profile and the Sano (SANO et al., 1976) and Puigdellivol (Puigdellivol et al., 1985) IM PK were best described using muscle blood flow 2–3.5 times lower than the default muscle blood flow (2.86 mL/min/100 g). In addition, while the core model parameters (pKa, Log P, PPB%, Rbp and specific permeability surface area product) combined with the Poulin and Theil extracellular tissue partition co-efficient (Kp) estimation method (Poulin and Theil, 2009, 2000) adequately describe tissue distribution for 6 out of the 7 studies evaluated this was not the case for the Puigdellivol dataset (Puigdellivol et al., 1985). To capture the slower IM absorption rate seen by Puigdellivol the muscle Kp was increased from the calculated value of 0.97 to 2.0, see Fig. 2 (IM; middle and IV; left panels). The increase in muscle Kp has only a minor impact on the overall Vss and shape of the predicted IV plasma profile however it does have a significant impact on the extracellular fraction of unbound drug in muscle (Fuext) which when dosing directly to the intramuscular compartment significantly reduces the unbound extracellular concentration of TXA at the site of injection thereby slowing the rate of systemic absorption.

### 3.1 Model performance

**Fig. 3:**
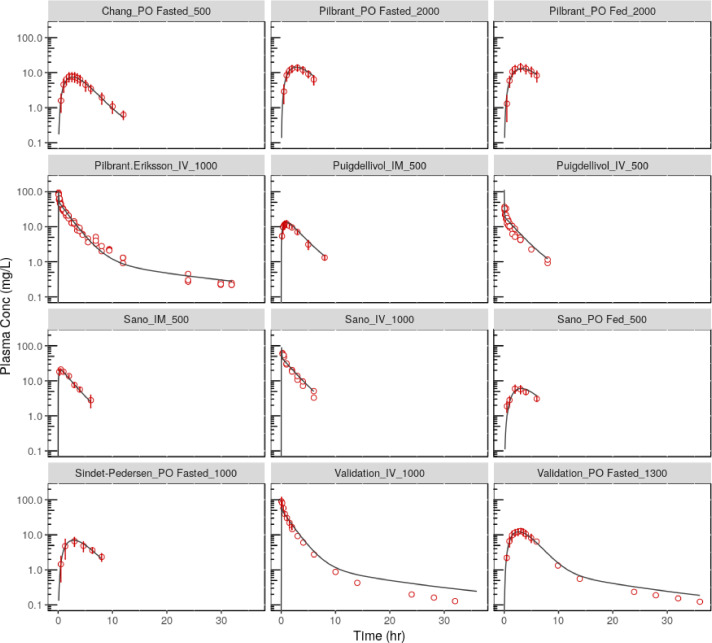
Model performance. Comparison of observed plasma concentrations (red open circles) with predicted levels (solid black line) for model development and validation datasets, see Table 1 for individual study details and Table 2, Table 3, Table 4, Table 5 for model parameters. Where appropriate and the necessary data was available, error bars show the standard deviation in plasma concentration at a given timepoint. (For interpretation of the references to colour in this figure legend, the reader is referred to the web version of this article.)

TXA plasma Cp-time profiles were simulated using the relevant parameters detailed in Tables 2, 3, 4 and 5. The full, predicted Cp-time profiles are shown compared to the observed data for each regimen evaluated in Fig. 3. The relevant summary statistics (Cmax, AUC, Tmax) and associated prediction errors (PE) are compared in Table 6. Across the populations, doses and routes of administration evaluated, the model performed with an average PE of 9 and 6% on Cmax and AUC0-t, respectively (Table 6). Considering the IV and oral validation dataset, both the predicted Cmax and AUC0-t are within 10% of the observed values (Xanodyne Pharmaceuticals Inc., 2009).

**Table 6:** Comparison of observed and predicted TXA pharmacokinetics across all datasets evaluated.

Source	Route	Ethnicity	Dose (mg)	Pred F%	Tmax (h)		Cmax (µg/mL)			AUC_0-t_ (µg*h/mL)			R^2^
					Obs	Pred	Obs	Pred	PE (%)	Obs	Pred	PE (%)	
Eriksson, Pilbrant	IV	European	1000		0.08	0.08	86.8	99.8	15	126.0	129.5	3	0.97
Puigdellivol 1	IV	European	500		0.08	0.08	33.4	25.7	−23	41.9	51.5	23	0.63
Sano 2	IV	Japanese	1000		0.26	0.07	59.8	88.6	48	104.0	111.9	8	0.78
Validation	IV	American	1000		0.05	0.08	90.4	95.2	5	120.7	126.1	4	0.83
Pilbrant (fed)	PO	European	2000	36	3.00	3.24	14.5	13.2	−9	61.3	58.2	−5	0.91
Pilbrant (fasted)	PO	European	2000	38	3.00	2.74	13.9	14.3	3	59.5	63.1	6	0.95
Sindet (fasted)	PO	European	1000	36	3.00	2.85	6.6	7.0	6	33.9	36.3	7	0.97
Sano (fed)	PO	Japanese	500	54	2.00	3.14	6.0	6.0	0	25.0	25.9	0.04	0.91
Chang (fasted)	PO	Chinese	500	67	2.53	2.64	7.8	7.5	−4	42.7	41.5	−3	0.98
Validation	PO	American	1300	52	3.07	2.64	12.9	12.2	−5	77.8	76.2	−2	1.00
Sano	IM	Japanese	500	99	0.50	0.44	21.1	20.4	−3	58.6	56.0	−4	0.97
Puigdellivol	IM	European	500	99	0.83	0.99	12.3	13.3	8	45.3	50.7	12	0.92
Average									4			4	

1 Administration was a bolus IV injection, therefore peak concentrations have been compared at the time of the first observation.

2 Infusion time and time of first plasma observation are inconsistent, predicted Tmax and Cmax reflects the source stated infusion time (4 mins).

Regarding the observed Cmax in the Sano et al. study, (SANO et al., 1976) and the high prediction error (48%), the infusion time is reported to be 4 min, however the first blood sample was not taken until *T* = 15 min. If a 15 min infusion time is assumed when simulating the Sano data the predicted Cmax is reduced to 59.8 µg/mL (0% Cmax PE).

### 3.2 PO Simulations

**Fig. 4:**
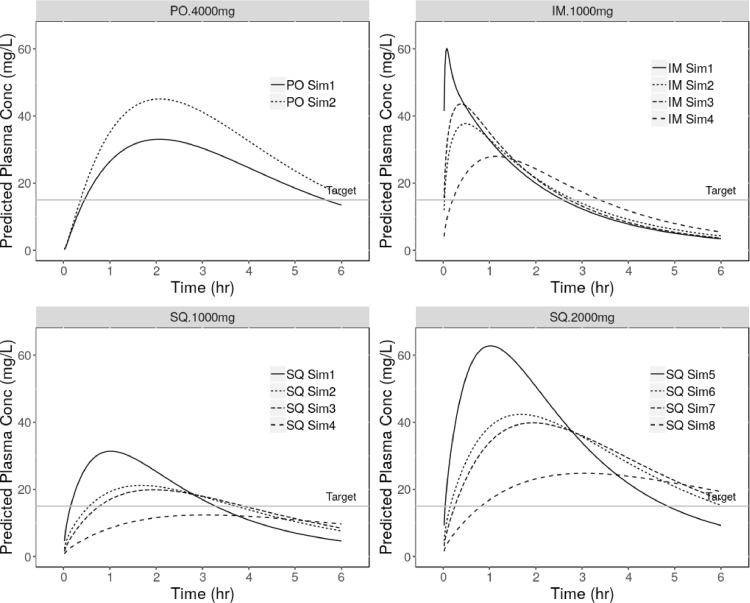
Simulation results. Different line types illustrate the predicted impact of; intestinal permeability (oral) and tissue blood flow and tissue partition coefficients (IM and SQ). For these key parameters, a range of estimates informed by model development were used in the simulations. See Table 7 and Section 3.2. for the corresponding simulation parameters. Top left - oral single dose 4000 mg fasted state. Top right - intramuscular single dose 1000 mg. Bottom left - sub-cutaneous single dose 1000 mg. Bottom right - sub-cutaneous single dose 2000 mg.

Two simulated oral Cp-time profiles (PO Sim1 and PO Sim2) are presented in Fig. 4 (top left panel), both assume dosing of 4000 mg as a solution (40 mL of a 100 mg/mL) administered in the fasted state. A stomach transit time of 0.1 h was used in both simulations, while PO Sim1 assumes a Peff of 0.4 × 10–4 cm/s and PO Sim2 a Peff of 0.5 × 10–4 cm/s. The permeability estimates used reflect (to 1.d.p) the highest and lowest values required to simulate the observed oral PK (Table 4 and Fig. 3) and for comparison the average Peff value of 0.45×10–4 cm/s was used when simulating the oral validation dataset (Xanodyne Pharmaceuticals Inc., 2009). The default human fasted advanced compartmental absorption and transit model (ACAT) was used and all other model parameters were as defined in Table 3 and Section 2.5.

The first oral simulation (PO Sim1; Peff = 0.4 × 10–4 cm/s) resulted in a Cmax of 33 µg/mL, AUC0-t 143 µg*h/mL and Tmax 2.1 h. The second simulation (PO Sim2; Peff = 0.5 × 10–4 cm/s) resulted in a Cmax of 45 µg/mL, AUC0-t 190 µg*h/mL and Tmax 2.0 h.

### 3.3 IM Simulations

IM Cp-time profiles were simulated for a solution dose of 1000 mg. The site of injection was defined as the gluteus. Due to software limitations, only a single 10 mL injection volume (of 100 mg/mL solution) could be simulated, rather than two 5 mL injections as would be clinically recommended, (Hopkins and Arias, 2013) thus resulting in a larger depot volume. In these simulations, the minimum calculated depot volume of 84.7 mL was employed.

Table 7 summarises for the four IM simulations (IM Sim1-IM Sim4) the estimates used for Q and Kp at the site of injection (gluteus muscle). These ranged from the default values for Q (2.86 mL/min/100 g muscle) and Kp (0.97) to a three fold lower estimate of Q (0.95 mL/min/100 g muscle) and 2 fold higher estimate of Kp (1.94), values informed by the model development work, see Table 5.

**Table 7:** IM and SQ simulation parameters and PK results .

Sim ID	Q at SOI (mL/min/100 g)	Tissue Kp at SOI	Depot Vol (mL)	Dose (mg)	Cmax (µg/mL)	AUC0-t (µg*h/mL)	Tmax (h)
IM1	2.86	0.97	84.7	1000	60	105	0.1
IM2	2.86	1.94			38	102	0.5
IM3	0.95	0.97			44	105	0.4
IM4	0.95	1.94			28	99	1.1
SQ1	2.6	0.97	74	1000	31	102	1.0
SQ2	2.6	1.94			21	90	1.7
SQ3	0.87	0.97			20	88	1.9
SQ4	0.87	1.94			12	62	3.1
SQ5	2.6	0.97	74	2000	63	205	1.0
SQ6	2.6	1.94			42	181	1.7
SQ7	0.87	0.97			40	176	1.9
SQ8	0.87	1.94			25	123	3.1

The resulting predicted IM PK parameters (Cmax and AUC) are also presented in Table 7. The simulated Cp-time profiles (IM1-IM4) are presented in Fig. 4 (top right panel) and show that even then slowest absorption rate (IM Sim4) plasma levels are predicted to exceed 15 µg/mL in ≤ 15 min. Concentrations are predicted to be maintained above the target level for between 2.5–3 h. Bioavailability via the IM route is predicted to be 100%, but the practical dose volume limitations mean the overall predicted AUC0-t (99 µg*h/mL) is 2.6 fold lower than the best case 4000 mg oral prediction; simulation PO2.

### 3.4 SQ Simulations

SQ Cp-time profiles were simulated for doses of 1000 mg initially but increased to 2000 mg in order to exceed the 15 µg/mL target concentration. In GastroPlus® the dose volume for a SQ administration is capped at 10 mL so for the 2000 mg simulation it was necessary to assume a 200 mg/mL solution of TXA was dosed, the feasibility of this concentration, from a formulation perspective, is unknown. The site of injection (SOI) was defined as adipose in the leg and as with IM dosing the same software limitations regarding the use of multiple injection sites applied. Therefore the minimum calculated depot volume of 74 mL was used in all SQ simulations.

Human PK data following SQ administration of TXA has never previously been published, therefore the same strategy developed for the IM simulations was employed; default software estimates and three fold lower for adipose blood flow (2.6 and 0.87 mL/min/100 g adipose) and default software estimates and 2 fold higher for adipose Kp (0.97 and 1.94). Table 7 summarises the adipose blood flow at the site of injection and adipose Kp used in each SQ simulation and the resulting predicted PK parameters (Cmax, AUC and Tmax). The simulated Cp-time profiles are presented in Fig. 4 (bottom panels).

## 4 Discussion

The present study describes the development of a PBPK model that can accurately describe single dose IV, oral (fasted and fed) and intra-muscular administrations of TXA (Chang et al., 2004; Eriksson et al., 1974; Pilbrant et al., 1981; Puigdellivol et al., 1985; SANO et al., 1976; Sindet-Pedersen, 1987). PK data from six different single dose studies performed in healthy volunteers were utilised during model development. External validation of the model was performed for the IV and PO routes of administration and prediction errors on Cmax and AUC0-t were ≤ 10% for both routes (Xanodyne Pharmaceuticals Inc., 2009). Across study variability in PK was captured through demographic (ethnicity, gender, age and body weight) effects on GFR, Vss and key absorption parameters specific to the route of administration. Intestinal permeability and stomach transit time were significant factors following oral dosing and muscle blood flow and partition coefficient were significant following IM dosing.

The range of values estimated by this model for total plasma clearance are compatible with TXA clearance values reported elsewhere in healthy volunteers and in cardiac surgery patients (Grassin-Delyle et al., 2013; Nilsson, 1980; Pharmacia and Upjohn Co, 2020; Pilbrant et al., 1981; Sharma et al., 2012).

No measured data was available on the blood to plasma ratio (Rbp) for TXA. Based on structure a Rbp of 1.35 was predicted using the ADMET software (version 7.2), however the data sets upon which these predictions are based are significantly smaller than for critical physical chemical properties such as Log P and pKa and higher variability in biological measures, such as Rbp, mean these predictions are not typically as accurate. The predicted value was tested during method development but a value of 1.0 better captured the observed volume of distribution.

A potential limitation of this work may result from the reduced number of participants involved in some of the source PK studies and a lack of associated demographic information. In addition no published PK data following SQ administration of TXA was found and therefore SQ simulations were performed by applying the same strategies as developed during modelling of observed IM PK data.

Other potential limitations include the fact that, apart from in the Sindet-Pederson study where 50% of the participants were female, (Sindet-Pedersen, 1987) all the participants in the model development studies were male, while the model validation study only included female subjects (Xanodyne Pharmaceuticals Inc., 2009). However for the validation dataset the predicted Cmax and AUC0-t were within 10% of the observed values, which does suggest that gender does not play a significant role in the PK of TXA.

Finally, despite an extensive search for all eligible PK data it is possible that some relevant studies may have been missed.

It is of note that using the Puigdellivol et al. data, the prediction error calculated for the IV AUC0-t was high, with the model over predicting the observed AUC0-t by 23%. This may be due to an under prediction of renal filtration in these participants which may be confounded by a lack of body weight information. The IV plasma levels reported by Puigdellivol et al. do stand out as lower and more variable than the other IV PK datasets evaluated during this work, however subject numbers in the Puigdellivol study were limited (*n* = 3) making it difficult to draw conclusions (Puigdellivol et al., 1985).

TXA is a high solubility compound, the oral bioavailability of which is limited by intestinal permeability. Oral bioavailability has been predicted to vary from 36 to 67% across the datasets evaluated and due to the absence of any significant first pass extraction in the liver or gut, oral bioavailability directly represents the fraction of drug absorbed (Fa). The oral bioavailability of the TXA regimens evaluated Pilbrant et al. were reported to be 33 and 34%, with our model predicting 36 and 38% respectively. Due to the low permeability of TXA even small differences in the effective human permeability (Peff) have been shown to have a significant effect on Fa . Whilst the high solubility of TXA means we would not expect formulation effects to limit the extent of exposure, certain formulation factors may influence the rate of absorption and while the European studies (Pilbrant et al., 1981; Sindet-Pedersen, 1987) used the same oral drug product (multiple 500 mg Cyklokapron tablets), the oral formulations used by Chang et al. and Sano et al. are unclear, as are the specific formulation details of the IR reference tablet dosed in the study XP12B-101 (Xanodyne Pharmaceuticals Inc., 2009).

After PO administration, plasma TXA concentrations are predicted to exceed 15 µg/mL within 15–30 min, to peak at between 30 and 60 µg/mL in 2 h and be maintained above 15 µg/mL for 5–6 h. The oral simulations presented here assumed rapid (0.1 h) gastric emptying of a solution formulation which is considered to be a reasonable assumption. Slower gastric emptying would result in slower absorption.

Comparitively there is more uncertainty associated with our IM predictions due to less data available for model development, significant variability in the two available datasets and the absence of an external validation dataset. Therefore, a larger number of parameter scenarios were evaluated (IM Sim1 to IM Sim4). All however predict that plasma concentrations exceed 15 µg/mL in ≤ 15 min when a 1000 mg dose is administered. Peak concentrations following a 1000 mg IM dose are comparable to the 4000 mg oral predictions, 30–60 µg/mL. The IM Tmax however is more ambiguous and a range of 0.1 to 1 h was predicted, with plasma levels falling below 15 µg/mL in 2.5 to 3.5 h. Muscle blood flow was identified as a very sensitive parameter during model development, and clinically is expected to be an important consideration as blood flow varies in different skeletal muscles (Evans et al., 1975; Vučićević et al., 2015).

Very little is known about the SQ use of TXA. Whilst there are reports of this route of administration being used for dermatological surgeries (Sagiv et al., 2018; Zilinsky et al., 2019) pharmacokinetic studies after SQ injection of TXA are not available. Based on our simulations SQ administration of TXA appears to be the route with the lowest potential for success using currently available formulation options. If a 2000 mg dose could be achieved through use of a higher concentration TXA solution SQ administration may become a more viable option. However, time to peak concentrations are still only either comparable to those predicted via the oral route, or slower with the most conservative parameters estimates (Table 7 and Fig. 3, simulations SC4 and SC8).

## 5 Conclusions

This analysis suggests further clinical evaluation of TXA administered via intra-muscular injection is warranted as it is predicted to be a valid alternative to IV administration for fast delivery of a therapeutic dose of TXA to the blood. High doses of TXA administered orally as a solution may also provide a valid administration method. However, studies including larger sample sizes are required to better describe the pharmacokinetics of alternative, but less characterized routes of administration of TXA, both in healthy volunteers and in emergency settings to account for traumatic situations such as hypovolemia.

## Data availability

All data analysed as part of this study came from previously published work and is available from the sponsor on request by emailing ctu@lshtm.ac.uk.

## Software availability

R is an open access statistical software freely available for download. GastroPlus®, ADMET® and DigitTM are proprietary software's produced by Simulation Plus, Lancaster, California, USA and are significantly discounted to support academic, not for profit research.

## CRediT authorship contribution statement

**Zoe Kane:** Conceptualization, Writing – original draft, Formal analysis, Data curation, Writing – review & editing. **Roberto Picetti:** Writing – review & editing. **Alison Wilby:** Writing – original draft, Writing – review & editing, Supervision. **Joseph F. Standing:** Writing – review & editing, Supervision. **Stanislas Grassin-Delyle:** Writing – review & editing. **Ian Roberts:** Data curation, Conceptualization, Funding acquisition, Writing – original draft, Writing – review & editing. **Haleema Shakur-Still:** Data curation, Conceptualization, Funding acquisition, Writing – original draft, Writing – review & editing.
